# Evolutionary Basis of Human Running and Its Impact on Neural Function

**DOI:** 10.3389/fnsys.2016.00059

**Published:** 2016-07-11

**Authors:** Jay Schulkin

**Affiliations:** Department of Neuroscience, Georgetown UniversityWashington, DC, USA

**Keywords:** running, brain, plasticity, neurogenesis, hippocampus, exercise, human

## Abstract

Running is not unique to humans, but it is seemingly a basic human capacity. This article addresses the evolutionary origins of humans running long distances, the basic physical capability of running, and the neurogenesis of aerobic fitness. This article more specifically speaks to the conditions that set the stage for the act of running, and then looks at brain expression, and longer-term consequences of running within a context of specific morphological features and diverse information molecules that participate in our capacity for running and sport. While causal factors are not known, we do know that physiological factors are involved in running and underlie neural function. Multiple themes about running are discussed in this article, including neurogenesis, neural plasticity, and memory enhancement. Aerobic exercise increases anterior hippocampus size. This expansion is linked to the improvement of memory, which reflects the improvement of learning as a function of running activity in animal studies. Higher fitness is associated with greater expansion, not only of the hippocampus, but of several other brain regions.

## Background

Fossil records indicate that we may have been proficient at walking upright as far back as 4.5 million years ago. Surviving skeletal remains show significant changes in our early ancestors’ upper and lower limb morphology. When we think of major evolutionary changes that affected the trajectory of our species’ development, bipedalism (followed by running) turns out to be a critical feature in our developmental history (Bramble and Lieberman, 2004). For instance, two of the original purposes of running may have been to follow wounded prey while hunting (Bramble and Lieberman, 2004) and to escape from predators. Indeed, specific aspects of running like speed as well as endurance—the ability to just keep going—were likely crucial for survival. In particular, human beings are very good at distance running. This may have evolved from a food-obtaining technique to a necessary social act associated with the spreading of news to one with recreational purposes. Today, running, in addition to other physical sports, is primarily performed for enjoyment and exercise. Despite the change in purpose, running can reveal and enhance our biological capabilities. Yet, running is, of course, not unique to humans. Other species, such as wolves and lions, are also both socially cooperative and have endurance.

In this article, I begin with some of the morphological conditions and physical adaptations that set the stage for the act of running. I then look at the role of metabolism and the brain, diverse information molecules that participate in our capacity for running and sport, several physiological features that underlie running (with particular attention to neurogenesis), and longer-term consequences of running.

## Morphology: What Makes Running Possible

We are socially cohesive predators, and lower-limb physical adaptations may have emerged about 2 million years ago to facilitate long-term trotting while tracking prey. Even though running and running in groups is not exclusive to humans, many paleoanthropologists now suspect that long-distance running may be a specific evolutionary adaptation to group hunting over long distances that evolved specifically with humans (Lieberman, 2011). Figure 2 demonstrates the effect of increasing toe length on peak digital flexor force in the lateral toes, which is an evolutionary adaptation that improves the capacity for running. The diverse skeletal features depicted in Table 1 are physical characteristics, such as long legs and short toes, that make long-distance running possible in our species (Bramble and Lieberman, 2004). Other related adaptations include an enlarged gluteus, a small waist, neck thorax flexibility, and expanded flexibility of vestibular and ocular reflexes.

**Figure 1:**
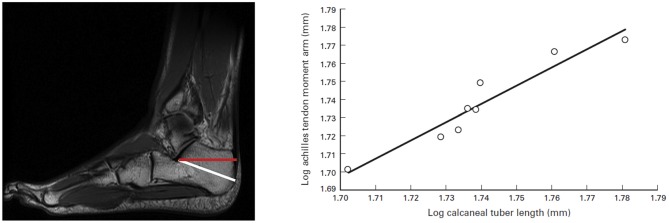
**Calcaneal tuber length (CTL) and moment arm measurements.** (Left) Sagittal MRI of foot and ankle. The white line is an approximation of the measurement for CTL, and the red line is the actual Achilles tendon moment arm. (Right) Relationship between CTL and Achilles tendon moment arm measurements. The correlation is significant (*r* = 0.95; *p* = 0.0002). The following ordinary least-squares regression line may be used to determine actual moment arm lengths from isolated calcanei (slope [95% confidence interval (CI)] = 1.00 [0.31]; intercept [95% CI] = −0.01 [0.54]; *r*^2^ = 0.91). Source: adapted from Raichlen and Gordon (2011).

**Figure 2:**
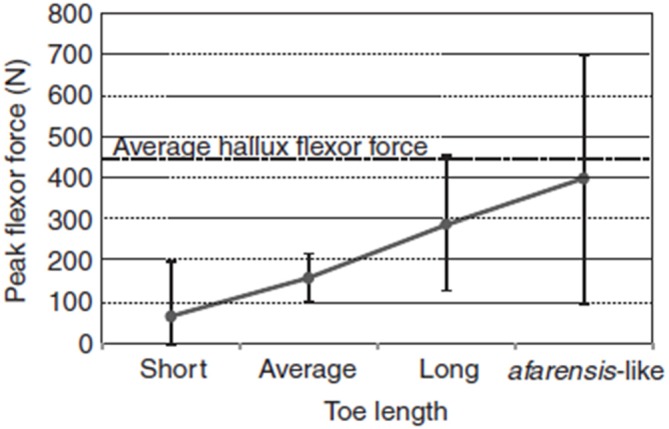
**Effect of increasing toe length on peak digital flexor force in the lateral toes, estimated using multiple regression equations derived from the study sample.** Dots and vertical bars represent the predicted peak forces and their 95% CI, respectively, for four hypothetical individuals having the same body mass, contact times and toe-out angle, but with toe lengths ranging between the sample extremes (Short, Average and Long) and scaled to A. *afarensis* lateral toe length (*afarensis*-like). The *afarensis*-like individual was obtained by increasing the average hallux length by 40% (although the hallux of A. *afarensis* probably was not that long). The dotted line represents the average digital flexor force in the hallux. Source: adapted from Rolian et al. (2009).

**Table 1:** **Derived features of the human skeleton with functions adapted for running**.

Feature	Functional role	Earliest evidence
More balanced head, short snout	Head stabilization	*H. habilis*
Tall, narrow body form	Thermoregulation	*H. erectus*
Forearm shortening, narrow thorax	Counterrotation of trunk	*H. erectus*
Narrow pelvis	Counterrotation of trunk vs. hips	*Homo?*
Stabilized sacroiliac joint	Trunk stabilization	*H. erectus*
Long legs	Stride length	*H. erectus*
Shorter femoral neck	Stress reduction	*H. sapiens*
Long achilles tendon, plantar arch	Energy storage	*Homo?*
Permanently adducted hallux	Stability during plantar flexion	*H. habilis*
Short toes	Stability during plantar flexion	*H. habilis*
	Distal mass reduction	

Diversification of the foot played a key role in the origins of locomotion and running. By the time *Homo erectus* emerged some 3.5 million years ago, a modern foot, almost indistinguishable from ours, had evolved (Lieberman, 2007). As seen in Figure 1, the calcaneus length is importantly tied to this evolutionary trend, facilitating a morphological design that favors efficiency in long-distance running. Long Achilles tendons and short toes are key morphological features of *Homo erectus* (Lieberman, 2012) that contributed to endurance capability in exploring and hunting and are associated with speed. Furthermore, the length and flexibility of the Achilles tendon is critical in warm climates for distance runners (Raichlen et al., 2013).

**Figure 3:**
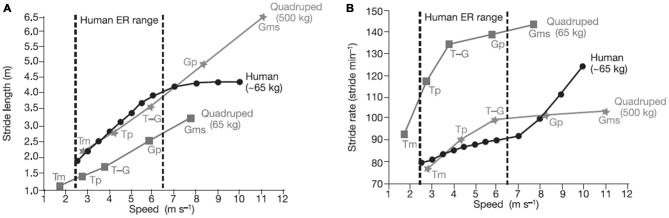
**Comparison of stride length (A) and stride rate (B) contributions to running speed in humans, and in quadrupedal mammals (calculated from ref. 25) for various gaits.** A stride is a complete locomotor cycle (two steps for a human). Compared with similar-sized quadrupeds, humans have relatively long stride lengths and relatively low stride rates in the endurance running (ER) range. Humans increase speed within the ER range primarily by increasing stride length, not rate. Source: adapted from Bramble and Lieberman (2004).

Bipedalism, which evolved over the last 5 million years, may have also aided movement in trees (Thorpe et al., 2007). Some primates and apes (e.g., chimpanzees) are bipedal for short bouts and in some contexts, but their hip, spinal, and limb structures do not make bipedalism an optimal mode of locomotion for long periods of time, and they are certainly not efficient runners. The subtle morphological changes that made it possible for *Homo erectus* to move bipedally also allowed it to enlarge its territory, which may be tied to an expanded brain and increased technological capability. Figure 3 shows how humans have relatively long stride lengths and low stride rates compared to four-legged animals of a similar size. Brain expansion led to greater cognitive/affective capacities and may have eventually resulted in the exploration and the development of technology and culture (and eventually sport). Environmental factors (e.g., climate) may also have contributed to bipedalism and brain expansion (Falk, 2004).

Efficient bipedalism requires a narrower pelvis than that found in australopithecines. For some species, bigger brains and a narrower pelvis meant more difficult parturition. *Homo erectus* females probably would have required assistance from others in giving birth, and their infants were likely born at an earlier stage of neural development than those of other primates. This has implications for hominin social structure, indicating a certain level of cooperative social behavior and an extended juvenile stage. Diverse hormonal developments also may be involved in human birth and parturition patterns. Of particular note, the nuclear progesterone receptor (NPR) gene might be involved in this evolutionary process, relating bigger brains with human pregnancy and parturition.

Running speed is, of course, tied to the length of the leg and the stride, within a context of conservation of energy and maximization of resources through the utilization of glucose and the maintenance of fluid volume and loss. Steroid hormones such as aldosterone, an adrenal steroid hormone, are tied to fluid volume and sodium conservation, which are essential in maintaining fluid levels, solute volume, and tonicity for continued viability (Denton, 1982). Thus, conservation at all levels is operative, as sodium and water excretion drop under conditions of long-distance running in the tropical climate in which humans evolved.

Perhaps our need to roam far is also related to our nutrition needs. We were and are meat-eaters, we are more accurately categorized as omnivores (Rozin, 1998). We can manage as vegetarians or even vegans—hundreds of thousands of human beings survive and even thrive on such diets. We can do quite well on a meat-only diet; Eskimo and Inuit have done so for thousands of years. But the range of nutrients and fiber we require is most easily satisfied by a combined meat-and-plant diet. The range of what hominins from *Home erectus* ate, and most definitely what we eat, reflects omnivory.

**Figure 4:**
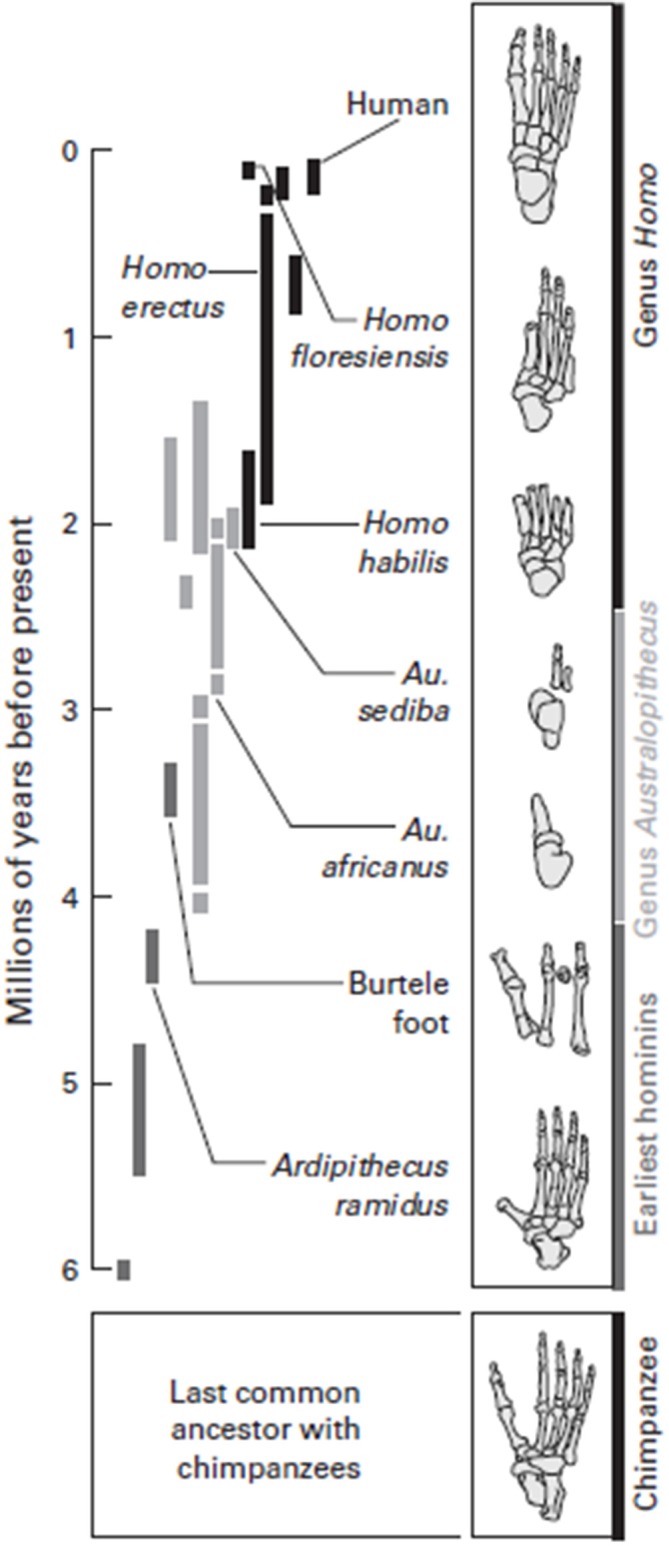
**Walking along the evolutionary tree.** Hominins have evolved many various forms of feet since diverging from the common ancestor they most recently shared with chimpanzees, about 6 million years ago. The early hominin species *Ardipithecus ramidus* was adapted for both walking and climbing trees, but, like a chimpanzee, had a highly divergent big toe and probably used its feet more like a chimpanzee than like a modern human when it walked. Foot fossils from more recent hominin species, such as the *Homo habilis* and *Homo floresiensis*, have a more complete arch. It was probably not until Homo erectus that very human-like feet evolved, with a completely developed arch and a large big toe aligned with the other toes. Feet adapted to both bipedal locomotion and tree-climbing persisted for a long time in human evolution. Source: adapted from Lieberman (2012).

Perhaps 3.5 million years ago, the feet of related hominoids indicate they may have both climbed trees and walked erect, with long toes, a grasping big toe, and a flexible arch. Bipedalism with such a foot would have been effective, but these hominins were likely not able to run distances as seen in Figure 4. Changes in the foot, as our hominin ancestors committed more and more to a ground-dwelling existence, set the conditions for our capabilities associated with running, coupled with the expansion of the cortex, which facilitated an increase in all our physical capabilities.

Bipedalism is a very important adaptation, and a human-like gait can be traced back to *Australopithecus africanus* some 4 million years ago. Additionally, the expansion of the shoulder muscle, the Achilles tendon, and the larynx (for speech and singing; Lieberman, 1984) are adaptations that set the stage for our modern-day social and physical engagement with sports.

## Metabolism

Our metabolism has the capacity for long-term movement while treading over great distances, and our exploration often required endurance while navigating through environments where excessive heat loss was a concern. Our ability to regulate our internal milieu to account for variable environmental conditions is yet another evolutionary adaptation that helped our ancestors continue to traverse great distances and harsh environments.

The metabolic costs of walking are similar across higher primates and therefore early hominins (Pontzer et al., 2009), but walking became less metabolically expansive with greater motor dexterity. Energy expenditure is a key evolutionary feature, and walking upright is tied to our evolution, since it allowed us to better see and maximize our resources. Standing and looking and efficiency are part of our evolution. Indeed, standing straight may have been a metabolic advance (McHenry, 1994).Walking, perhaps by the middle of the Pliocene for hominins, was more efficient and less metabolically demanding than the ground locomotion of other kinds of apes (Pontzer et al., 2009).

When one compares the efficiency of locomotor expenditure (Pontzer et al., 2009), selection pressure favored short hind legs and long arms in chimpanzees, because that sort of structure is more efficient for getting up and moving around in trees (Wrangham, 1987; Pontzer et al., 2009), vs. our upright position. The above figure demonstrates the evolution of the hominin foot over the last six million years; the evolution of the hominin foot has facilitated the human’s capacity to walk and run.

Metabolic rates and regulation of running and walking are key features, since our ancestors moved from one area to another. Sheer physical capability and brain size are correlated to one another (Raichlen and Gordon, 2011), and metabolic rate serves as an indicator of the regulation events essential toward this end.

The brain is an active organ. A great massive absorption of glucose is required to sustain its energy expenditure, the activity of the neuronal assemblage that underlies its diverse activities. Indeed, glucose utilization is a core feature of metabolic regulation in every organ system and all cells, but particularly the brain; glucose utilization is a marker of brain activity and can be used to mark relationships, for instance between memory and learning. Furthermore, glucose utilization maintains functional architecture in the brain, networks that underlie skill in sports or memory and learning. Brain activation may account for roughly 70% of energy demands in the brain (Dunbar and Shultz, 2007). Aerobic exercise increases anterior hippocampus size. This expansion is linked to the improvement of memory, which reflects the improvement of learning as a function of running activity in animal studies. There appear to be region-specific differences (e.g., expansion of the hippocampus) in the level of exercise in primates. As running evolved from a solitary action of following wounded prey while hunting and escaping from predators to a more social function of running in packs, as wolves are known to do.

In other words, the evolution of the brain is tied to physical capability, running being one feature of this evolution. Indeed, metabolic rate is a feature of athleticism, as are brain mass and physical capability. One of the more interesting findings in recent years, however, is the correlation between physical or exercise capacity and the resulting brain size in diverse mammals (Raichlen and Gordon, 2011).

Diverse studies have demonstrated the links between brain activation and glucose metabolism in the brain and other end organ systems. Thus the size of the brain region and the expanded utilization of glucose under normal conditions facilitate an expansion in energy expenditure and allows for running.

## Endorphins and Other Information Molecules

A major discovery, during the age in which many peptide hormones were discovered, was that of the endorphin hormones. The possibility that chemical signaling included messages that reduced pain or were tied to the highs that are associated with being on drugs was speculated, and then confirmed.

Diverse information molecules are tied to neural plasticity, and all have properties that are growth factors. Researchers uncovered a common chemical messenger in growth factors (a diverse family of peptides) that underlie developmental trajectories (the Nobel Prize for uncovering this went to Rita Levi-Montalcini and Stanley Cohen in 1986). Some of these growth factors include Activin, Colony Stimulating Factor, Connective Tissue Growth Factor, Epidermal Growth Factor, Erythropoietin, Fibroblast Growth Factor, Galectin, Growth Hormone, Hepatocyte Growth Factor, Insulin-Like Growth Factor Binding Protein, Insulin, Insulin-Like Growth Factor, Keratinocyte Growth Factor, and Leptin.

The endorphins consist of a large group of peptides that date back at least a half billion years. They are expressed in both vertebrates and invertebrates, in which they are diversified in expression through various end organ systems (e.g., brain, pituitary) and other peptide hormones. These peptides, like many others, were discovered in the brain during the great revolution in biochemistry during the 1970’s and 1980’s, when many neuropeptides were discovered (Skofitsch and Jacobowitz, 1984).

It is still not completely clear what the roles of these diverse endorphins are, but they range from the reduction of pain to euphoria to positive links associated with social attachment (Koob and Le Moal, 2005, 2008). One of course wants to distinguish diverse endorphins expressed in the central nervous system (CNS) from the peptides expressed in the brain itself and those expressed in the periphery, and the peptides detected in extracellular fluids from those detected in the cerebrospinal fluid. A blood brain barrier keeps (BBB) peptides in the periphery.

Endorphins in the CNS are site-specific in terms of whether they enhance pleasure, and it has been found that endorphin injections induce pleasure. Moreover, endorphin-like substances in the periphery are either elevated or reduced during withdrawal, and not necessarily elevated during drug ingestion or pleasure. But most forms of psychoactive substances result in the elevation of endorphin-like substances (Skofitsch and Jacobowitz, 1984).

One important neurotransmitter is, of course, dopamine. The interaction of dopamine and endorphin-like substances may underlie part of the good experience that is tied to long-distance running: the transmitters, perhaps, for long-term stability and the neuropeptide for fleeting moments of euphoria.

Euphoria is reported in many studies of running. Running can be viewed as a way of reducing stress-related events, as are other forms of physical exercise. Diverse studies over a number of years have found endorphin levels in runners to be elevated. Use of PET to measure endogenous endorphins both before and after a 2-hour run found dramatic changes in the expression of endogenous endorphins in the binding of the radioactive ligand (Boecker et al., 2008).

There are visible changes in the opoidergic binding in neurons in the frontal areas, limbic/paralimbic areas, temporoporietal areas of the brain when there are changes in the Visual Analog Scale (VAS) ratings of euphoria (Boecker et al., 2008).

Of course, it’s not all running highs and happy feelings. Long-distance running partially involves combating pain and discomfort, common themes in many sports. Adversity is inherent in sport. To struggle is to succeed, and to cope with struggling, the human body has evolved to release hormones associated with euphoric states so that when one is faced with a particularly trying physical feat, the cephalic space is permeated with chemicals that induce a sense of calmness, and what seemed daunting ceases to be much of a bother at all.

This would implicate both central and peripheral physiological signaling systems to induce the states of quiescence, of “no worries”. Some runners do experience periods of sheer intensity, but a sense of having no worries predominates. Another theme is a sense of accomplishment. Having achieved a perceived goal is one feature of a runner’s high. During sports of any kind, the body is being pushed to its limit and often beyond it, but the euphoria that comes with great physical exertion delivers the reward needed for continued physical pursuit regardless of the exhaustion and pain otherwise associated with that activity.

Pleasure is not one thing, after all, but a grab-bag of states. Various forms of effort and relief are thus linked to different information molecules acting in diverse ways in shared brain regions.

Regions of the brain—including the nucleus accumbens, a region of the basal ganglia tied to the organization of movement—are responsive to information signals, including those of endorphins or opioids (Peciña et al., 2006). The interactions of these two regions, the nucleus accumbens and the central nucleus of the amygdala, represent a link between motor and motivation via a common type of information molecule: endorphins.

## Endocannabinoids

The endogenous endocannabinoid is tied to the stress response, the sense of adversity that underlies animal activity in general and human’s playing sports in particular. The reduction of endocannabinoid signaling is an adaptation to adversity, as evidenced by the management of the HPA axis (Hill et al., 2010). The breakdown of this signaling system contributes to negative states. Genetically reared mice that are without this endocannabinoid signaling ran half as much as they would normally—and they normally run a lot.

One view of the endocannabinoids is that they are protective under conditions of adversity (Hill et al., 2010). Long-distance running is but one example of information molecules playing protective roles in long-term tissue viability.

Exercise-induced elevated levels of endocannabinoid (eCBs) signaling occur in humans and several other species (Raichlen et al., 2013). In humans, treadmill running and intensity are linked to the endocannabinoid signaling. The idea is that eCBs is tied to reinforcing properties of “runner’s high” (Raichlen et al., 2013). We also know that a variety of other information molecules are altered in the brain via running behavior (neurotrophic factors, for instance).

The expansion of the neocortex (e.g., cell type, neural connectivity) in mammals and its resulting organization is associated with various physical behaviors in animals (Elston, 2007; Krubitzer, 2007). Interestingly, exercise capability is linked to a feature of cortex expansion (Raichlen and Gordon, 2011), which is not what one typically thinks of with cortical expansion.

**Figure 5:**
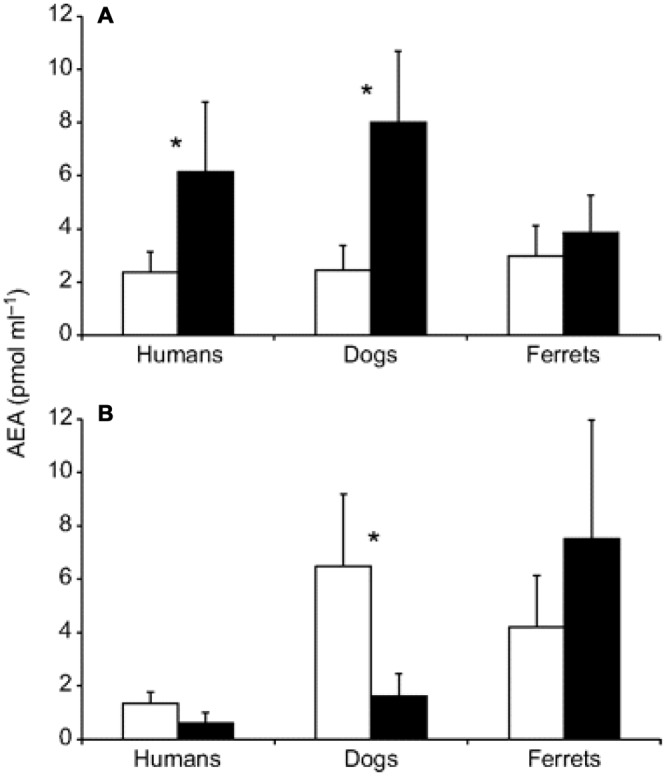
**Changes in anandamide (AEA) concentrations before and after treadmill exercise.** Pre-exercise levels are shown in white; post-exercise levels are shown in black. **(A)** Plasma AEA levels before and after running for 30 min at a Froude number of 0.70. **(B)** Plasma AEA levels before and after walking for 30 min at a Froude number of 0.25. Asterisks indicate significant differences at *P* < 0.05. Error bars are SEM Source: adapted from Raichlen et al. (2012).

Cannabinoid receptors, such as anandamide (AEA), are linked to reward signaling, but that description of purpose is probably too narrow. What we do know is that these information signals are expressed and regulated during sports such as running. Figure 5 summarizes acomparative study in humans, dogs, and ferrets, where cannabinoid receptors were more elevated after running than after walking (Raichlen et al., 2013).

**Figure 6:**
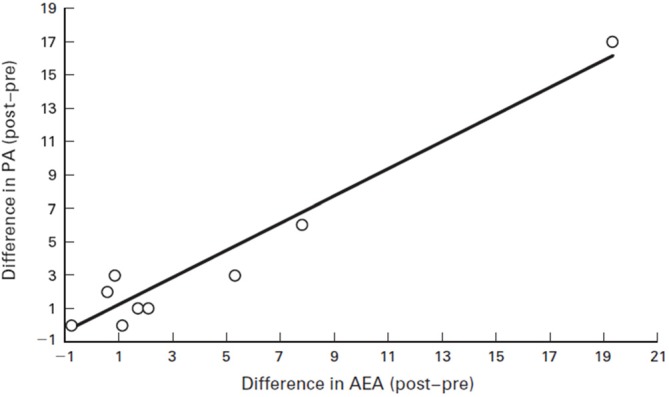
**Correlation between positive affect (PA) and AEA in humans.** Values are the difference between pre- and post-exercise PA scores plotted against the difference between pre- and post-exercise plasma levels of AEA. Note that the values for two subjects were nearly identical (difference in AEA = 0.89 and 0.90, difference in PA = 3 for both subjects), and they are not differentiated on the figure. Source: adapted from Raichlen and Gordon (2011).

For instance, humans who had an AEA concentration of 2.2 pmol/ml before running had that concentration jump to 6.1 pmol/ml after running for 30 min at a Froude number of 0.70. The Froude number was used to calculate the walking speed in humans when body mass differences were taken into account. The Froude number was calculated by [velocity2/(hind limb length gravitational acceleration) ≈0.25 (Raichlen et al., 2012). After walking for 30 min at a Froude number of 0.25, humans who started with an AEA concentration of 1.7 pmol/ml saw their AEA concentration sink to 0.9 pmol/ml (Raichlen and Gordon, 2011). In Figure 6, Raichlen and Gordon (2011) demonstrate the positive correlation between positive affect (PA) and AEA in humans.

## Genetics and Epigenetics

Genius and capability are tied to genes and circumstance, temperament and opportunity. Very few of us can match Tom Brady, Joe Montana, or Tiger Woods—great athletes like this are really geniuses of a sort (Zimmer, 2005). Their raw ability, the astonishing focus they bring to bear to turn that ability into excellence, and the sheer ingenuity of their anticipatory skills are dazzling (Babiloni et al., 2010).

**Figure 7:**
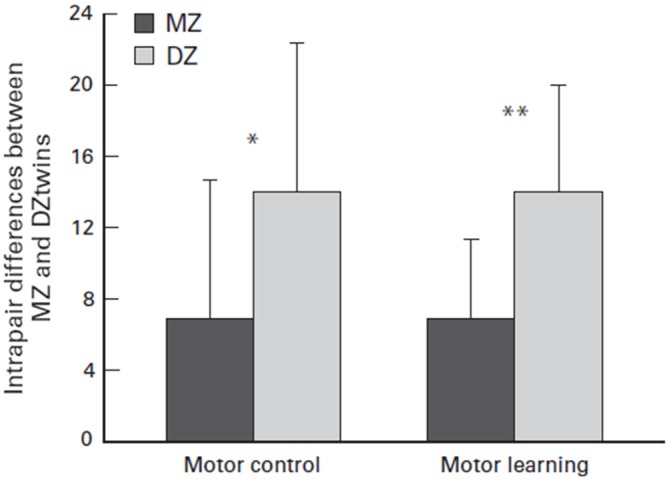
**Mean and standard deviation (SD) of intrapair differences between monozygotic (MZ) and dizigotic (DZ) twins in motor control and motor learning.** Asterisks indicate significant differences (paired *t* test; *P = 0.05 and **P = 0.01, respectively). Source: adapted from Missitzi et al. (2013).

Their raw capacity may largely be a matter of genetic luck. Several twin studies have explored the factor of genetic luck when the studies concluded that there was a genetic link to physical capability or athleticism. For example, Bouchard (2012) monozygotic (MZ) twin study concluded that there may be a strong genotype dependency for exercise ability due to the heritability of maximal oxygen uptake response. Furthermore, as seen in Figure 7, Missitzi et al. (2013) twin study suggests that heredity may explain some of the extant differences in motor control and motor learning between pairs of MZ and dizygotic twins. Both of these studies affirm the idea that genetics play a strong role in an individual’s athletic capability due to the mediation of biological factors that are partially determined by genetics, such as maximal oxygen uptake response, motor control and motor learning.

Mustelin et al. (2012) study demonstrates this link by finding that the heritability for sports activity is 64%. But capability has to be coupled with endless training and a supportive social context, a family that encourages and sustains, and as the cliché goes, the way to get to Carnegie Hall is to practice, practice, practice. We all know that talent goes only so far, and in the end not very far at all when one is in the major leagues. The world is littered with talent that has never been fulfilled. Discipline, perseverance, support, opportunity, and what used to be called character are needed to fulfill the promise of talent.

Epigenetics are the changes in DNA methylation due to a given subject’s activity and life experience. Such changes are tied to both genes and the regulation of genetic changes in practice and sport. Histone modification is one cornerstone of modern epigenetic research.

Endurance in running is a key feature in our evolutionary success as a species (Lieberman, 2007). Endurance is enhanced by the effects of exercise and lifestyle, but a number of genes, including nuclear respiratory factors, hemoglobin, and skeletal muscle glycogen synthase, contribute to human endurance abilities. Several of these are growth factor compounds (e.g., insulin), and a number of others are transmitters (adrenergic receptor b3).

Several genes have been linked to endurance. These genes are also tied to epigenetics changes, another of which is the angiotensin-converting enzyme; angiotensin is tied to fluid balance (Denton, 1982; Fitzsimons, 1998).

Physical activity, like other forms of human action, directly impacts genomic expression (Booth et al., 2002). For example, culture may change our biology and may make such changes possible via sport; the changes in both hypothetical genetic and epigenetic changes, which reflect a growing field in the analysis of sports.

Neurologically, enhanced attention is tied to the narrowing of neuronal assemblies; there is less neural expenditure and enhanced, focused neural activity. That is the adaptive side of neuronal assembles; consider the focus of the archer, the pitcher, the goalie. Zooming in with unremitting focus is necessary, but still not sufficient for sports excellence.

One mechanism that may underlie epigenetic gene regulation is methylation and demethylation (e.g., silencing or enhancing the expression of genes; Holliday and Ho, 1998). Demethylization prevents transcriptional expression, and one result is the silencing of gene expression. Such events in biological structure underlie sports capability and the shaping of the brain through training and practice. For instance, genes that produce oxytocin expression are particularly significant. This important peptide hormone plays diverse regulatory roles, from lactation to parturition to social attachment, depending on where it is expressed in end organ systems (Carter et al., 1997/1999).

## Brain, Running, and Neurogenesis

Exercise affects diverse regions of the brain. Aerobic exercise increases anterior hippocampus size (Chaddock et al., 2010; Erickson et al., 2011). Raichlen and Gordon’s (2011) study is also linked to the improvement of memory, which reflects animal studies on the improvement of learning as a function of running activity. Higher fitness is associated with greater expansion, not only of the hippocampus, but of several other brain regions.

Children who participate in aerobic activity or fitness have enhanced cognitive capabilities (Chaddock et al., 2011). Moreover, basal ganglia volume is associated with more fit adolescents (Chaddock et al., 2010). The effects are usually not overwhelming but are consistent. We do not know about causation from these studies.

There is a large literature on animal studies linking neurogenesis to diverse regions of the brain (McEwen, 2007; Paredes et al., 2016). This link to neurogenesis is not unique to humans. The hippocampus in particular is perhaps the best known and most studied structure in which neurogenesis—the generation of neurons from neural stem cells and progenitor cells—occurs (Gould, 2007). The hippocampus is a region of the brain primarily linked to memory (Squire, 2004).

Studies using mammals have shown us that running in a running wheel is strongly linked to hippocampus neurogenesis and that diverse peptides, such as brain derived neurotrophic factor (BDNF) are linked to both running and neurogenesis and recovery. BDNF is one of the many peptides that are tied to sustaining tissue fundamental in adaptation and sport, and to regions of the brain such as the hippocampus.

Running in animal models have consistently found cell proliferation and neurogenesis in the dentate gyrus (van Praag et al., 1999), in which diverse growth factors underlie the cell proliferation and neurogenesis (Vivar et al., 2013). Such running induces neurogenesis and promotes the formation of memory and learning (Shors et al., 2001).

**Figure 8:**
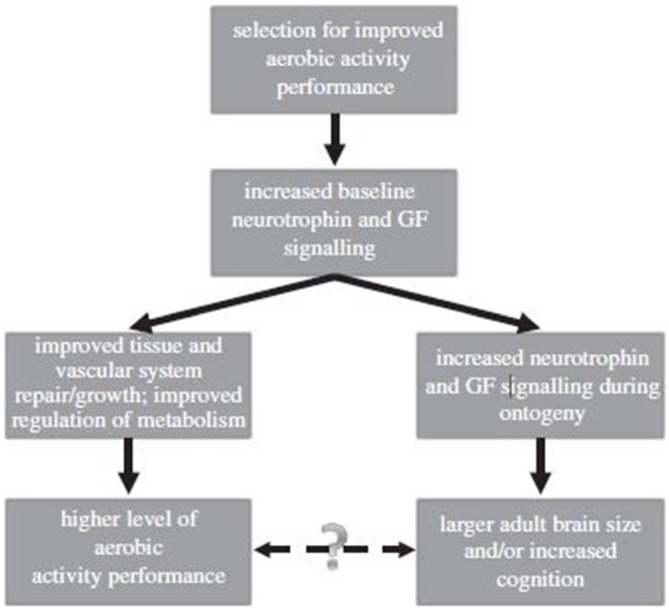
**Bilateral hippocampal volume as a function of aerobic fitness group.** Error bars represent SEM. Source: adapted from Chaddock et al. (2010).

Perhaps such events are lifelong. Aerobic capability or fitness is also associated with hippocampal volume in older individuals (between 60 and 70 years old) and their ability to perform memory tasks (Erickson et al., 2009). Thus, increases in volume expansion in both younger and older adults are associated with aerobic exercise. Clearly, aerobic activity is beneficial both in childhood and for older people, and brain expansion and memory capability are tied to aerobic activity levels as seen in Figure 8 (Chaddock et al., 2011). Indeed, plasticity in the brain as a function of exercise is quite remarkable as a phenomenon of nature. For instance, the bilateral hippocampal volume in groups with lower levels of fitness was around 6850 mm^2^, but around 7800 mm^2^ in groups with a high level of fitness (Chaddock et al., 2010).

Drugs such as Selective Serotonin Reuptake Inhibitors, or SSRIs, promote neurogenesis in the dentate gyrus (Pinnock and Herbert, 2008), but so does physical exercise in animal models. In animal studies physical exercise can prevent the reduction of hippocampal neurogenesis and cognitive capability following chemotherapy (Wincour et al., 2014). In human studies aerobic exercise can promote neurogenesis and ameliorate opposing effects of depression (Déry et al., 2013).

## Conclusion

Running is an expression of our evolution and importantly tied to neural expression. Diverse morphology set the conditions for running, and physiological signals facilitate adaptation and increased performance in running as well as other sports. Of course, speed and size interact with shape and endurance and neural capabilities.

Additionally, neurogenesis is a feature of several regions of the brain, most notably the hippocampus. Running-induced activity facilitates the expansion of the hippocampus in a number of species.

In other words, running itself promotes cell proliferation in the hippocampus, in part through the induction of endorphins or diverse neuronal growth factors (Koehl et al., 2008). Running and neurogenesis are linked to forms of basic adaptation; running easily transitioned from joint coordination to play, and eventually to sport (Grégoire et al., 2014). Diverse information molecules, particularly growth hormones and endorphins (Koehl et al., 2008), facilitate and sustain the expression of neurogenesis in regions of the brain such as the hippocampus during running activity.

Most important is the clear link from walking to running to sport, and the social context within a context of appraisal systems. In general, and in many sports, we are chronically appraising events, such as in our ability to infer strength from sounds.

Athletic capability, effort and exercise, physical play and sport all reveal a positive impact on neural function. In athletics specifically and in life more generally, striving, desiring, and succeeding are constant throughout our lifespan.

## Author Contributions

JS is the sole contributor to this manuscript. He was responsible for the manuscript conception and authorship. The author confirms being the sole contributor of this work and approved it for publication.

## Conflict of Interest Statement

The author declares that the research was conducted in the absence of any commercial or financial relationships that could be construed as a potential conflict of interest.
